# Case of Pericarditis, Connected with Acute Rheumatism

**Published:** 1827-12

**Authors:** S. D. Broughton

**Affiliations:** Senior Surgeon to the St. George's and St. James's Dispensary, and Surgeon to His Majesty's second Regiment of Life Guards.


*Case of Pericarditis, connected with acute Rheumatism, successfully treated* by S. D. Broughton, Senior Surgeon to the St. George's and St. James's Dispensary, and Surgeon to His Majesty's second Regiment of Life Guards.

The case of which I am about to give an account, though one now well known and understood, may nevertheless be regarded as a useful addition, perhaps, to the general stock of information upon the subject to which it relates.

It will be observed that the metastatis of acute rheumatism was the obvious exciting cause of an inflammatory affection of the heart, that the alternations of the disease in the different structures which it involved were strongly marked and clearly traceable, and that the mind was affected in a singular manner during its progress.

The case terminated favorably; which, in conjunction with its unequivocal signs, I deem to be not the less interesting to the pathologist on that account.

A recruit of the Life Guards, recently from the country, aged nineteen, tall, but of rather a slender form and pallid complexion, was taken into the regimental hospital on the evening of the 3d of April, 1826. He complained of pains throughout the body, principally referred to the joints. The tongue was white, the pulse full and quick, the skin dry, and the bowels were moderately free.

I prescribed seven grains of James's powder with three of Calomel; and placed him on spoon-diet, with barley-water and nitre.

April 4th.—The skin appeared moist, but the action of the pulse was little diminished.

One drachm of the Vinum Antimonii Tart. was given in a saline draught every six hours.—The powder was repeated, and an aperient draught of salts and senna ordered in the morning.

5th.—A slight abatement of the fever seemed to have taken place, and the pains were diminished.—Pergat.

6th.—Some degree of inflammation of the pharynx.

A gargle of Nitrate of Potass prescribed, and the mixture repeated.—Calomel and Antimony as before.

7th.—Some increase of fever, and return of pains in the joints, occurred today, and he was put into a hot bath. The antimonial wine excited too much nausea.

The dose was decreased, and the mixture given every four hours, with ten grains of James's powder every six.—The Calomel omitted.

Soreness of the pharynx relieved.

Next day, he appeared to be much relieved, with moisture on the skin, and less fever.

9th.—He experienced great pain and tumefaction of the knee and ankle on one side, with extreme tenderness to the touch. Bowels open ; tongue furred.

I ordered half a drachm of the Colchicum Wine to be given with each dose of the mixture, and ten grains of the compound powder of Ipecacuanha, with six of James's powder, every six hours ; and a fomentation of poppies to be applied to the limb.

10th.—The pain and swelling of the limb was much relieved, and now an urgent cough and fixed pain at the sternum appeared. The tongue was very dry and furred ; the pulse hard, rapid, and full. A deep inspiration excited pain and coughing, indicating acute inflammatory action, probably in the pericardium. The respiration did not seem to indicate the lungs as the seat of the disorder.

Twenty-four ounces of blood were directly taken from a large orifice in the arm ; the Colchicum was omitted, and the Antimony increased to double the former dose.—Ten grains of James's powder, with two of Calomel, were given in the evening ; and thirty leeches applied to the chest,

—the bleeding in the morning not having removed the pain, although it had diminished the fever, and the heart being felt acting powerfully against the ribs. The blood taken in the morning was much cupped and buffy.

A blister was applied to the back, between the shoulders, at night.

11th.—Some relief from the urgent symptoms of the day previous seemed to be obtained, but the action of the heart and arteries continued high and strong. The tongue was more moist, and less furred. The wrist of the left arm was swollen, red, and tender, and there was pain in the shoulder when moved. He had rather a restless night, but the skin was moist. The application of the leeches was followed by a disposition to syncope. The cough was considerably diminished, and the bowels were open. The pulse the evening before was at 130, but diminished in strength. The urine was very turbid, but clear this morning; and the swelling of the limb went down after the application of poppy fomentations, but remained stiff.

Twenty drops of the Tincture of Digitalis were added to each dose of the mixture.

12th.—Slept well last night. Some pain in the left arm. Pulse ninety-six; tongue moist and cleaning. Very little cough, and pain of the chest much diminished. Feels weak and low.

The Digitalis was increased to twenty-five drops every five hours.

Today he was allowed a little broth, and the following draught was given to remove a retention of urine from the blister, viz.

Misturæ Camphor, Misturæ Salinæ, āā ℥j.; Træ. Hyoscyami ʒss.; Spirit. Ætheris Nitrosi ʒjss. M. st. sum.

13th.—Passage of urine free; bowels open. The cough was nearly gone; pulse reduced to eighty-four. Slept well. Tongue moist and clean; the left arm much better; skin moist.

14th.—This morning the cough had somewhat increased, but the pulse continued at eighty-four. Complained of ardor urinæ. for which twenty drops of the Liquor Potassæ were given in barley-water and solution of gum arabic occasionally, together with ten grains of the compound powder of Ipecacuanha every six hours.

15th.—Pulse eighty-four. The ardor urinæ relieved, and the urine, which was thick yesterday, is now clear. The bowels were open, and he was better in every respect.

The Digitalis omitted.

16th.—The urine was scanty and turbid; the blister healed.

Pergat.

17th.—Urine clear and freer. Much debility.

Pergat. The antimonial wine omitted.

19th.—Pain of the chest and cough returned; pulse rose to 120, and rather full; tongue dry.

Twenty-four ounces of blood taken from a large orifice in the arm.—Diet reduced to spoon. The mixture, with one drachm of antimonial wine, repeated every four hours. The Dover's powder omitted. A large blister applied to the chest. A moderate draught of Salts and Senna given.

In the evening, he was very much relieved. The blood appeared much cupped and buffy.

20th.—The pulse was much reduced, and he was better, and continued so, but weak, till the 25th, when a fixed pain in the left side came on, not increased by a deep inspiration. The pulse rose to 116, but without cough.

Twenty-four leeches were applied to the side, and twenty drops of the Tincture of Digitalis added to each dose of the mixture.—One drachm of Colchicum-wine was given at night.

26th.—In the evening he was much relieved, and slept during the night. The pain was removed, but was disposed to return at intervals. The pulse was quick, but diminished in strength; respired freely, and had no cough; skin moist.

The Colchicum was repeated this night, and the mixture continued every six hours.

27th.—Pain returns periodically in the region of the heart; the pulse was bounding and intermittent, and the heart acted forcibly against the ribs, as if struggling in a confined space, with a sensation of fluttering in the chest. When on his back he complained of suffocation, and respired best in an upright position. Slept well last night. The tongue was moist and clean. No stool today.

The Colchicum omitted. The mixture continued, and thirty drops of the digitalis given at each dose. An opening draught.

28th.—Last evening his pulse became reduced in strength and frequency, and he was low and restless.—Pergat.

29th.—Pulse sixty, and did not intermit; urine high-coloured and clear; restless. This day he appeared confused in his mind, and talked incoherently.—Pergat.

30th.—The incoherency continued, and *he repeated the last word of every question and remark made to him.* The extreme fulness and force of the heart's action somewhat lessened.

The mixture repeated, and the blister renewed between the shoulders.

May 1st.—He appeared to have recovered his self-possession, and to have nearly left off repeating the last word addressed to him. Slept better last night, and was more composed. He respired easily: the pulse was less full; the heart beat less forcibly, but the artery pulsated irregularly. Tongue moist and clean, and bowels open.

During the few following days, he appeared to improve, but rambled a little, and sometimes the repetition of the last word was observable, and his spirits appeared low. The pulse was reduced to fifty-six, and the heart's action diminished. The artery at the wrist intermitted in its beats. Latterly his mind seemed less affected.—On the 4th, the Digitalis and Antimonial Medicine was omitted, and a draught of Infusion of Roses, with Epsom Salts, given thrice a day; and his diet was rendered more generous, in a small degree.

9th.—The intermission of the pulse continued, but less so, and he seemed to complain only of debility, with less restlessness and mental affection.

19th.—Has sat up every day, and, when upright, the pulse beat steady and at 100, but not strong or full. Night sweats have come on, but he has slept well, without pain or cough. The heart's action continued quick and forcible. Since the 16th, he had taken a grain of Digitalis with three of the Pil. Hydr. twice a day, and yesterday the Digitalis was increased to a grain and a half. The following pills were now prescribed—

Digitalis gr. v.; Hydr. Subm. gr. v.; Extracti Conii ʒj. M. fiat pil. xv. quarum sumat[r]; ter in die.

An ointment of one drachm of Tartarised Antimony, rubbed up with an ounce of the Ung. Cetacei was applied every night to the chest, till the pustules produced rendered its omission necessary.

He continued to gather strength, and the pulse became slow, natural, and equable; and on the 24th of June he left the hospital, and returned to his duty, having been under treatment since the 3d of April.

He has since continued perfectly well, and has never relaxed from his duties a single day. On examining him recently, I found the heart seemed to labour against the ribs: but his health was excellent, and his constitution in good condition, his conduct being steady, sober, and quiet.

